# Blocking connexin 43 and its promotion of ATP release from renal tubular epithelial cells ameliorates renal fibrosis

**DOI:** 10.1038/s41419-022-04910-w

**Published:** 2022-05-31

**Authors:** Huzi Xu, Meng Wang, Yinzheng Li, Mengxia Shi, Zheng Wang, Chujin Cao, Yu Hong, Bin Hu, Han Zhu, Zhi Zhao, Xiaoxin Chu, Fan Zhu, Xuan Deng, Jianliang Wu, Fenfei Zhao, Jing Guo, Yuxi Wang, Guangchang Pei, Fengming Zhu, Xiaoyan Wang, Juan Yang, Ying Yao, Rui Zeng

**Affiliations:** 1 Division of Nephrology, Tongji Hospital, Tongji Medical College, Huazhong University of Science and Technology, 1095 Jiefang Avenue, Wuhan, 430030 China; 2 Wuhan Institute of Biotechnology, Wuhan, 430000 China; 3 Wuhan Biobank, Wuhan, 430000 China; 4 Department of Urology, Tongji Hospital, Tongji Medical College, Huazhong University of Science and Technology, 1095 Jiefang Avenue, Wuhan, 430030 China; 5 Department of Nutrition, Tongji Hospital, Tongji Medical College, Huazhong University of Science and Technology, 1095 Jiefang Avenue, Wuhan, 430030 China

**Keywords:** Extracellular signalling molecules, Cell death

## Abstract

Whether metabolites derived from injured renal tubular epithelial cells (TECs) participate in renal fibrosis is poorly explored. After TEC injury, various metabolites are released and among the most potent is adenosine triphosphate (ATP), which is released via ATP-permeable channels. In these hemichannels, connexin 43 (Cx43) is the most common member. However, its role in renal interstitial fibrosis (RIF) has not been fully examined. We analyzed renal samples from patients with obstructive nephropathy and mice with unilateral ureteral obstruction (UUO). Cx43-KSP mice were generated to deplete Cx43 in TECs. Through transcriptomics, metabolomics, and single-cell sequencing multi-omics analysis, the relationship among tubular Cx43, ATP, and macrophages in renal fibrosis was explored. The expression of Cx43 in TECs was upregulated in both patients and mice with obstructive nephropathy. Knockdown of Cx43 in TECs or using Cx43-specific inhibitors reduced UUO-induced inflammation and fibrosis in mice. Single-cell RNA sequencing showed that ATP specific receptors, including P2rx4 and P2rx7, were distributed mainly on macrophages. We found that P2rx4- or P2rx7-positive macrophages underwent pyroptosis after UUO, and in vitro ATP directly induced pyroptosis by macrophages. The administration of P2 receptor or P2X7 receptor blockers to UUO mice inhibited macrophage pyroptosis and demonstrated a similar degree of renoprotection as Cx43 genetic depletion. Further, we found that GAP 26 (a Cx43 hemichannel inhibitor) and A-839977 (an inhibitor of the pyroptosis receptor) alleviated UUO-induced fibrosis, while BzATP (the agonist of pyroptosis receptor) exacerbated fibrosis. Single-cell sequencing demonstrated that the pyroptotic macrophages upregulated the release of CXCL10, which activated intrarenal fibroblasts. Cx43 mediates the release of ATP from TECs during renal injury, inducing peritubular macrophage pyroptosis, which subsequently leads to the release of CXCL10 and activation of intrarenal fibroblasts and acceleration of renal fibrosis.

## Introduction

Renal interstitial fibrosis (RIF) is a pathological disorder characterized by the excessive deposition of extracellular matrix material and the proliferation of fibroblasts in the renal interstitium [1, 2]. RIF has been identified as the common route by which all forms of chronic kidney disease (CKD) progress to end-stage renal disease [3]. Once RIF occurs, it is irreversible [4]. Therefore, exploring the mechanism(s) underlying RIF development may lead to effective therapies to prevent CKD progression, which is an urgent clinical need.

It has long been accepted that renal tubule injury is the ultimate endpoint of kidney disease [5]. Moreover, increasing evidence has shown that renal functional decline is more tightly correlated with tubular injury than with glomerular injury [6, 7]. However, renal tubular epithelial cells (TECs) are now considered not only the sites of injury, but also as key pro-inflammatory and fibrogenic cells that promote the progression from acute to chronic kidney disease [8, 9]. After injury, TECs release pro-inflammatory factors and recruit inflammatory cells, aggravating kidney damage [9–11]. Thus, preventing such functions by TECs is a potential avenue to delay RIF.

Gap junction proteins (GJs) are a type of cellular junction channels, which act as a hemichannel to transmit signals and death information to neighboring cells [12–14]. GJs are upregulated after kidney injury in humans and rodents [15–18]. Although GJs have been reported to be involved in renal disease, our understanding of the involvement of GJs in renal pathophysiology is limited [19–24]. Among these GJs, connexin 43 (Cx43) is the most common member [25, 26]. It has been identified that the development of cardiac fibrosis, liver fibrosis, and the acute phase of lung injury in patients are correlated with Cx43 expression [27–33]. Heqing Huang et al. demonstrated that the expression of Cx43 prevented the progression of diabetic kidney disease [34]. However, Chadjichristos et al. determined that the progression of CKD is delayed after Cx43 is systemically blocked [15]. So, the role of Cx43 in renal fibrosis has not been fully identified. Cx43 is widely distributed in various types of kidney cells. As TECs are essential in the maintenance of kidney function, the role of Cx43 in TECs attracted our attention [16, 19].

Cx43 can facilitate ATP release in various tissues under physiological and pathological conditions [35–38]. In this study, we genetically depleted and pharmacologically inhibited Cx43 to explore its role in RIF. We found that Cx43 promoted the release of ATP from injured TECs, which in turn induces the pyroptosis of macrophages surrounding these epithelial cells, thus activating an inflammatory reaction that promotes renal fibrosis.

## Results

### The expression of Cx43 is positively correlated with the severity of renal injury

**Fig. 1:**
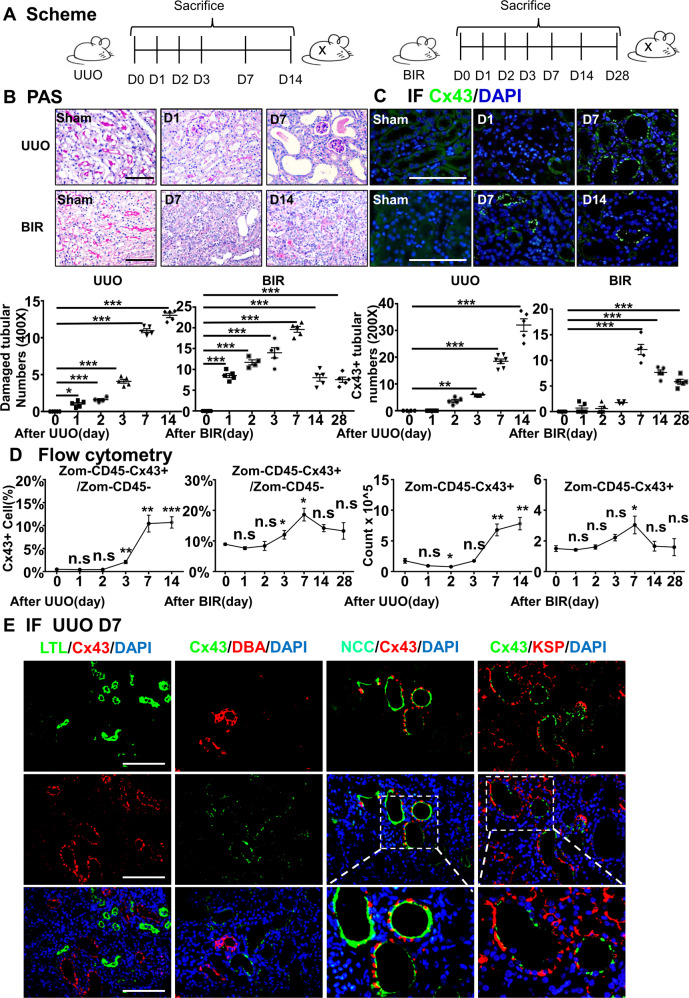
The expression of Cx43 correlates with the severity of kidney injury. **A** Scheme. time-related comparison after UUO and BIR. **B** PAS staining. PAS staining was executed to evaluate renal tubular injury by calculating damaged tubules. **C** Immunofluorescence. Cx43-positive tubules were counted at different points. **D** The percentage (left) and number (right) of Cx43-positive tubules analyses by flow cytometry from the whole kidneys. **E** Immunofluorescence. The immunofluorescence co-staining of NCC (a collecting tubule cells specific marker), KSP (a renal tubular epithelial cell-specific marker), LTL (a proximal tubule cells specific marker) or DBA (a distal tubule cells specific marker) with Cx43. Data are presented as the mean ± SEM, *n* = 4–7/group, *P < 0.05, **P < 0.01, ***P < 0.001; Scale bar: 100 μm.

Numerous studies have shown that GJs control the flow of metabolites across the epithelial layer, and thus they play an indispensable role in renal physiological and pathological process [39–42]. In this study, we measured the expression of Cx43 in kidneys after UUO and BIR injuries at different time points (day 1 (D1), day 2, 3, 7, 14 and 28) (Fig. 1A). We observed exacerbated tubular injury over time as well as continuously increased expression of Cx43 located in TECs (Fig. 1B, C). Flow cytometry gate strategies (Supplementary Fig. S1A, B) and analysis revealed that both the percentage and count of Cx43-positive cells increased in kidneys after UUO and BIR (Fig. 1D), with a similar tendency as that in immunofluorescence staining (Fig. 1C). This finding was further confirmed by RT-qPCR and WB (Supplementary Fig. S1C, D, and E).

As the Cx43 expression and specificity were higher in UUO on Day 7, we chose the model and the timepoint for further experiments. We detected the exact location of Cx43 in UUO kidneys. Immunofluorescence staining of LTL (a proximal tubule cells specific marker) and DBA (a distal tubule cells specific marker) along with Cx43 showed no regions of co-staining. The co-staining of NCC (a collecting tubule cells specific marker) or KSP (a renal tubular epithelial cell-specific marker) with Cx43 presented co-localization (Fig. 1E), implying that Cx43 is located in the collecting duct segment.

### Pharmacological blockade of Cx43 hemichannel attenuates UUO-induced renal injury and RIF

**Fig. 2:**
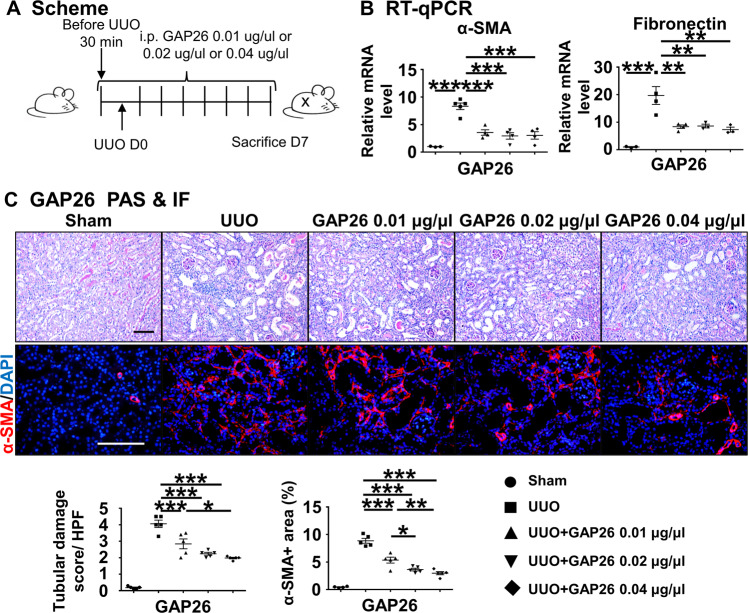
Cx43 hemichannel blocker attenuates kidney injury and RIF after UUO. **A** Scheme. mice were treated with GAP26 by i.p. injection after UUO surgery. **B** RT-qPCR. Levels of mRNA encoding α-SMA, Fibronectin by RT-qPCR. **C** PAS and immunofluorescence staining. PAS staining was performed to evaluate renal tubular injury by calculating tubules about GAP26. α-SMA is a marker of myofibroblast. Corresponding graphs indicate the expression of α-SMA for evaluation of myofibroblast infiltration. Data are presented as the mean ± SEM, *n* = 4–7/group, *P < 0.05, **P < 0.01, ***P < 0.001; Scale bar: 100 μm.

To further understand the role of Cx43 in renal injury and fibrosis, we applied the specific Cx43 hemichannel blockers, GAP26 after UUO surgery (Fig. 2A). RT-qPCR showed that the expression of α-SMA and of fibronectin in the GAP26-treated group was significantly lower compared to the untreated UUO group (*P* < 0.05) (Fig. 2B). PAS staining showed that there were massive renal tubule expansion, along with epithelial cell brush border loss, in the UUO group, while in the UUO + GAP26 group the number of injured tubules was lower (*P* < 0.05). Immunofluorescence staining of α-SMA, a marker of myofibroblasts and fibrosis, showed that numerous α-SMA-positive cells appeared in the renal interstitium in the UUO group, while in the GAP26-treated groups the number of α-SMA-positive cells was lower (*P* < 0.05) (Fig. 2C). These results suggest that blocking Cx43 hemichannel prevents UUO-induced renal injury and RIF.

### Cx43 gene knockdown in TECs prevents UUO-induced renal injury and RIF

**Fig. 3:**
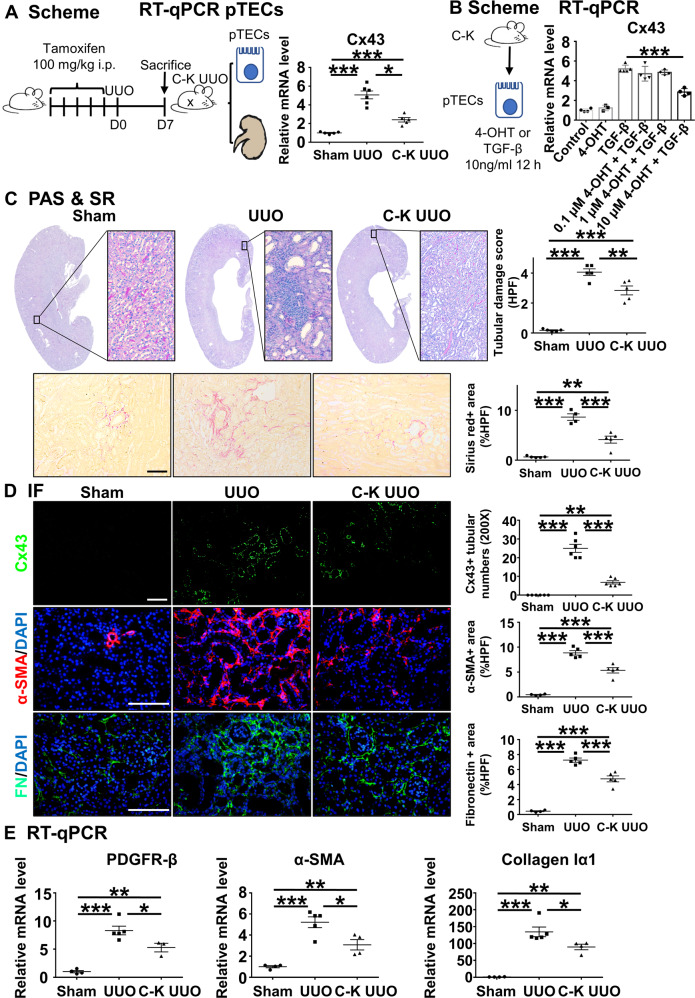
Knockout the Cx43 gene of TECs prevents UUO-induced renal injury and RIF. **A**, **B** Experiment schematic and Levels of mRNA encoding Cx43 by RT-qPCR. **C** PAS staining was performed to evaluate renal tubular injury by calculating damaged tubules with dilation swelling or epithelial cell brush border loss. The red area in SR staining indicates extracellular fibrotic collagen deposition in renal interstitium. **D** Immunofluorescence. Representative photomicrographs for immunofluorescence labeled Cx43 (green), α-SMA (red), and FN (green). Corresponding graphs indicate the expression of Cx43 (green), α-SMA (red) and FN (green). **E** RT-qPCR. Levels of mRNA encoding α-SMA, Fibronectin, Collagen Iα1 by RT-qPCR. Data are presented as the mean ± SEM, *n* = 3–6/group, *P < 0.05, **P < 0.01, ***P < 0.001; Scale bar: 100 μm.

Cx43 plays an important role in normal physiology, and thus systemic depletion of Cx43 expression causes serious complications, such as arrhythmia [15, 43, 44]. Therefore, to clarify the role of Cx43 in renal injury and fibrosis, we generated tubular-specific Cx43 knockout mice Cx43^fl/fl,Ksp-icre^ named C-K mice (Supplementary Fig. S2A, B). The morphology of organs excluding kidney showed no pathological change between the UUO and C-K UUO groups (Supplementary Fig. S2C). In vivo, Cx43 expression was knocked down by injection of tamoxifen, and then these mice were subjected to UUO surgery (herein, referred to as C-K UUO mice). We isolated primary TECs (pTECs) from C-K UUO mice and wild type mice injected by tamoxifen with UUO or not. RT-qPCR found the expression of Cx43 in the C-K UUO mice was significantly lower compared to the UUO. (Fig. 3A). Transforming growth factor β (TGF-β) has been identified as the main inducer of epithelial-to-mesenchymal transition and thus fibrosis in the kidney [45]. In vitro, the pTECs from C-K mice were cultured with 4-OHT, an active form of tamoxifen, to knockdown Cx43, and simultaneously treated with TGF-β. We found the most effective concentration of 4-OHT was 10 μM, in which the expression of Cx43 was lower compared with the TGF-β group (Fig. 3B). These data further confirmed the successful construction of Cx43^fl/fl,Ksp-icre^ mice. PAS staining showed that in the C-K UUO group the number of injured tubules and the tubular damage score per high-power filed (HPF) were lower. Sirius Red (SR) staining showed the percentage of the deposition of fibrotic collagen in the whole field was lower in the C-K UUO group (4.14% HPF) compared to the UUO group (8.67% HPF) (Fig. 3C). After intraperitoneal injection of tamoxifen, the expression of Cx43 showed about a 72.67% ((25-6.83)/25%) reduction in the C-K UUO group compared with the UUO group, implying successful knockdown of Cx43 expression in TECs (Fig. 3D and Supplementary Fig. S2D). Next, we stained for α-SMA and found an 8.86% α-SMA-positive area in the UUO group compared to 5.16% in the C-K UUO group. Fibronectin staining showed a similar tendency, in which the percentage of the fibronectin-positive area declined from 7.26% in the UUO group to 4.76% in the C-K UUO group (Fig. 3D). RT-qPCR confirmed the down-regulated expression of renal fibrosis-related genes, including PDGFR-β, α-SMA, and Collagen Iα1, in the C-K UUO group compared to the UUO group (Fig. 3E). These data suggest that Cx43 gene knockdown in TECs prevents UUO-induced renal injury and renal fibrosis.

### Knockdown of Cx43 expression influences the purine metabolism pathway in TECs

**Fig. 4:**
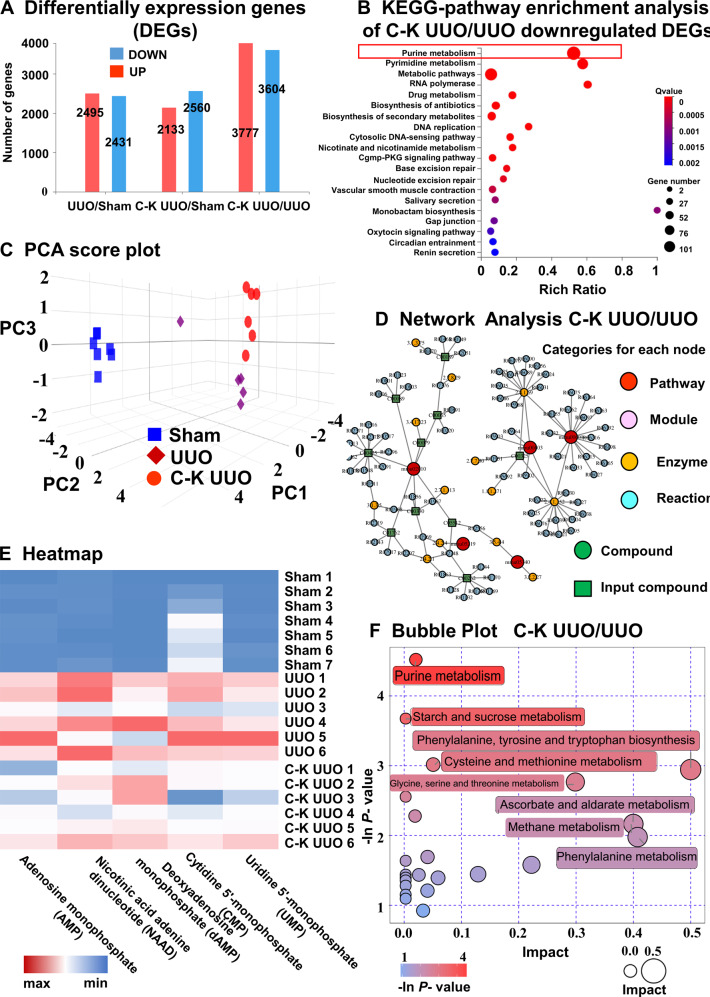
Transcriptomics and Metabolomics of C-K UUO mice revealed that knockout of Cx43 gene mainly affects the accumulation of purine substances in TECs. **A** Differentially expression genes. **B** KEGG-pathway enrichment analysis of C-K UUO/UUO downregulated DEGs. **C** Principal component analysis (PCA) score plot. **D** Network analysis of differential products between C-K UUO and UUO group. **E** Heatmap of selected enriched terms (FDR ≤ 0.01) from KEGG-pathway analysis of downregulated DEGs in C-K UUO group and upregulated DEGs in UUO group. **F** The bubble plot shows that the main difference between UUO and C-KUUO group is purine metabolites.

To explore the mechanism by which Cx43 promotes renal injury and fibrosis, we extracted pTECs from Sham, UUO and C-K UUO mice for transcriptome RNA sequencing (RNA-seq). The whole RNA-seq analysis showed that pTECs from the C-K UUO group had 3604 down-regulated genes compared to the UUO group (Fig. 4A). These genes were mostly involved in purine metabolism by KEGG-pathway enrichment analysis (Fig. 4B). We further analyzed the metabolome of urine samples and also found that the differential metabolites between the UUO group and C-K UUO group were mostly related to purine metabolism (Fig. 4C–F). Furthermore, Cx43 knockdown reduced the levels of purine metabolites in the urine, suggesting Cx43 participates in the regulation of purine metabolism in TECs. As a hemichannel, Cx43 responds to various external stimuli by mediating the release of adenosine triphosphate (ATP), glutamate, NAD+ and prostaglandin E2, in which ATP is the major source of purine-related metabolites [18, 23, 46, 47]. Therefore, as Cx43 expression is associated with worse UUO-induced renal fibrosis and the main metabolite released from the injured TECs is ATP, we hypothesized that this release is a major mechanism by which Cx43 promotes RIF.

### The Cx43 hemichannel on TECs regulates ATP outflow for binding to P2X7 receptor participates in renal fibrosis

**Fig. 5:**
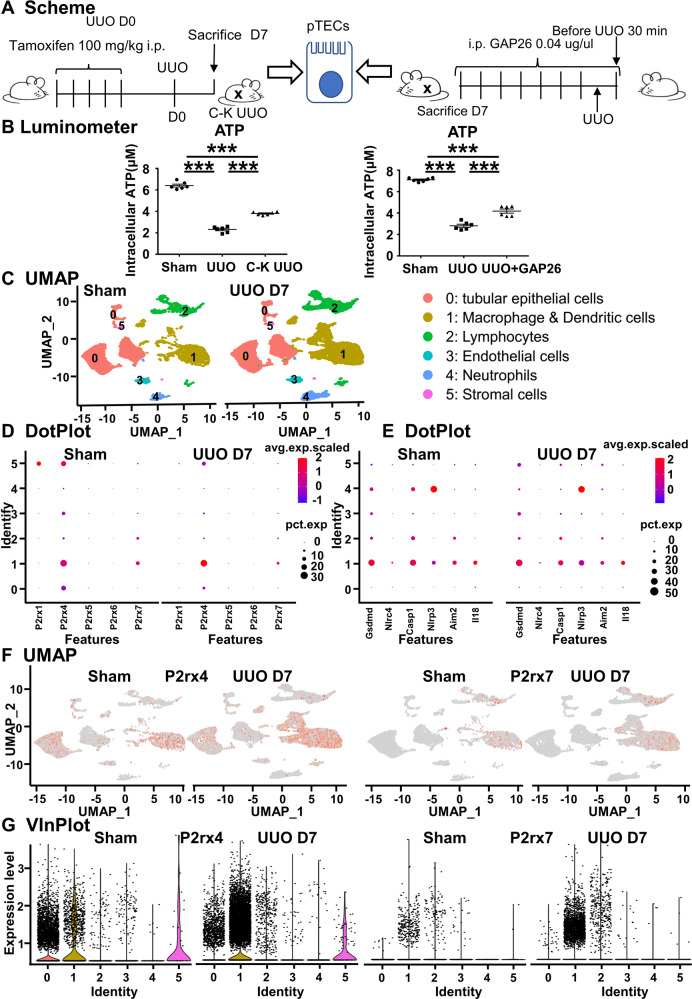
The Cx43 hemichannel of TECs mediate ATP outflow and ATP receptor analysis. **A**, **B** Detection of intracellular ATP content in TECs. **C** scRNAseq identified clusters of cells in the Sham and UUO kidneys after surgery at day 7. UMAP plot representation of 30,788 kidney cells, including 20,590 cells from UUO kidney and 10,198 cells from Sham kidney. DotPlot (**D**, **E**), UMAP (**F**) and violin plots (**G**) depicted genes related to ATP receptor and pyroptosis. Data are presented as the mean ± SEM, *n* = 4–7/group, ***P < 0.001.

To explore this possibility further, we extracted pTECs from the UUO, C-K UUO and UUO + GAP26 groups and then measured their intracellular ATP concentrations (Fig. 5A). We found that compared to the Sham group, the intracellular ATP concentration was lower in the UUO group, while the concentration was higher in the C-K UUO group compared to UUO group, implying knockdown of Cx43 in TECs prevented the outflow of ATP to the extracellular space. A similar change was observed after using GAP26 (Fig. 5B), further suggesting the outflow of ATP in TECs is dependent on Cx43.

Previous reports showed that ATP mainly binds to purinergic receptors on the surface of target cells to mediate cell proliferation, migration, differentiation and death [48–51]. According to the scRNA-seq cell clustering and marker genes, we divided all cells into six groups: TECs, macrophage & dendritic cells, lymphocytes, endothelial cells, neutrophils, and stromal cells (Fig. 5C). ATP receptors include P1 and P2 (P2X and P2Y). The P2X receptor is an ATP-specific receptor, including P2X1 to P2X7 [52]. Among P2X1 to P2X7, we observed that P2X4 and P2X7 are the ones most highly expressed in the kidney, mainly in renal macrophages (Fig. 5D). As the activation of P2 receptors is related to the activation of inflammasomes and pyroptosis, we analyzed pyroptosis-related genes and found that they were mainly located in macrophages (Fig. 5E). Jeffrey John Bajramovic et al. suggested that ATP-induced IL-1β secretion in BMDM was fully dependent on P2X7 signaling [53]. From UMAP and VlnPlot plots, we also found that the P2X7 receptor gene was specifically upregulated in macrophages after UUO (Fig. 5F, G). Thus, we assumed that ATP-associated macrophage pyroptosis might play an important role in renal fibrosis via ATP outflow from TECs.

To explore the effect of tubular ATP outflow and its receptor activation on renal injury and fibrosis, we used a P2 receptor inhibitor (suramin) [54] or a P2X7 receptor inhibitor (A-839977) [55] (Supplementary Fig. S3A). PAS and SR staining showed that compared with the UUO group, the tubular damage score was lower and fibrotic area was larger in the UUO + A839977 or +suramin group (Supplementary Fig. S3B, C). In addition, we found that treatment with a P2X7 receptor agonist (BzATP) [56] induced worse UUO-induced renal injury compared with the untreated UUO group (Supplementary Fig. S3D–F). These data indicate that the P2X7 receptor participates in UUO-induced renal fibrosis.

### The ATP outflow from TECs increases macrophage pyroptosis

**Fig. 6:**
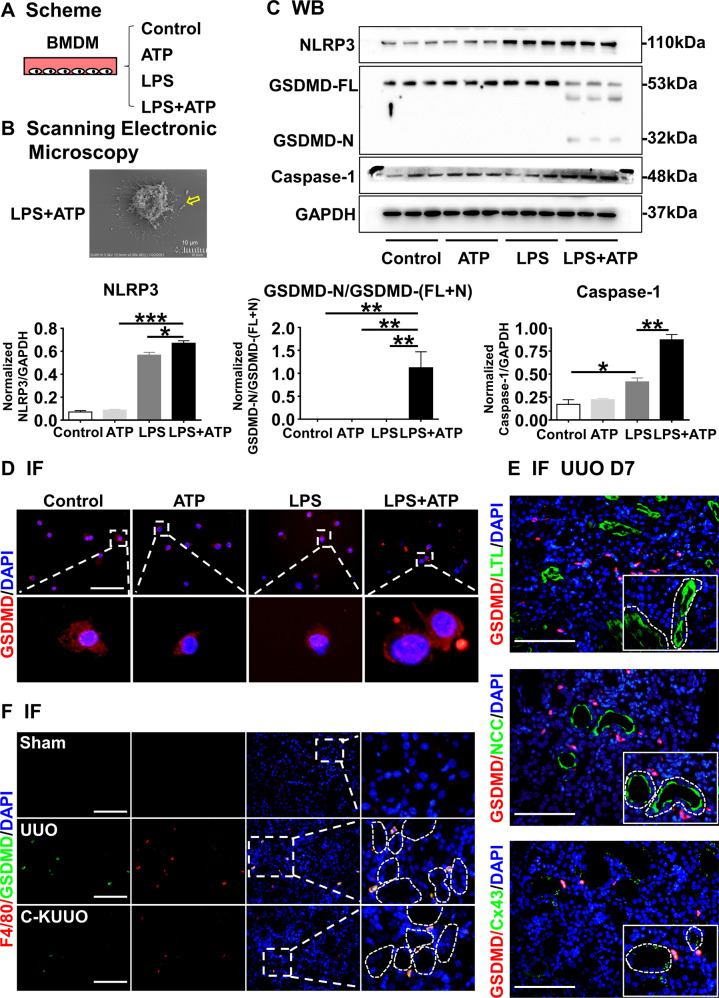
The ATP outflow from TECs increase macrophage pyroptosis via P2 receptor. **A** Scheme. BMDMs were primed with or without LPS (500 ng/ml) for 4 h, followed by stimulation with ATP (5 mM) for 6 h. **B** Representative scanning electronic microscopy (SEM) images of BMDM cells treated with LPS and ATP. Yellow arrow points to bubbling of pyroptotic cells. Scale bar, 10 µm. **C** LPS with or without ATP induction upregulated the protein expression of NLRP3, caspase1. LPS with ATP upregulated the protein expression of GSDMD-N/GSDMD-(FL + N). Corresponding graphs indicate the expression of NLRP3, caspase1, GSDMD-N/GSDMD-(FL + N). **D**–**F** Immunofluorescence. BMDMs Representative photomicrographs for immunofluorescence labeled GSDMD and DAPI of BMDMs indicated that LPS with ATP could active macrophage pyroptosis (**D**) and the pyroptotic cells reduced after knockout the Cx43 gene by immunofluorescence labeled GSDMD, F4/80 and DAPI (**E**). The pyroptotic macrophages were mainly concentrated around Cx43-positive TECs (**F**). Data are presented as the mean ± SEM, *n* = 3–6/group, *P < 0.05, **P < 0.01, ***P < 0.001; Scale bar: 100 μm.

A recent study reported that ATP activates inflammasomes after binding to P2X7 receptors [57]. In addition, it has been suggested that the activation of inflammasomes is closely related to immune cell pyroptosis [58]. In Fig. 5E, we observed the genes related to the inflammasome (Nlrc4, Nlrp3, Aim2, Casp1) and pyroptosis (Gsdmd, Il18) were mainly expressed in macrophages. To investigate the specific effects of ATP on the macrophage pyroptosis, we induced cellular pyroptosis in bone marrow-derived macrophages (BMDMs) by stimulating them with LPS and ATP (Fig. 6A). Pyroptosis was observed by scanning electronic microscopy, as marked by characteristic swelling, nuclei concentration and the emergence of vesicle-like pyroptotic bodies, in LPS + ATP-treated cells (Fig. 6B). WB analysis showed that NLRP3, Caspase-1, GSDMD-N and GSDMD-FL (GSDMD-full length) were markedly activated after LPS + ATP stimulation. However, these proteins were not significant changes when BMDM cells were stimulated with LPS or ATP alone (Fig. 6C). Immunofluorescence staining demonstrated that LPS + ATP induced the translocation of GSDMD-N towards the plasma membranes, forming pyroptotic bodies and activated cell pyroptosis (Fig. 6D).

Next, we extracted pTECs from mice in the Sham group and the C-K group, and co-cultured them with BMDMs. The extracellular ATP concentrations of BMDM were regulated by an ATP analog (ATPγS) or an ATP-depleting agent (Apyrase). RT-qPCR revealed that the expression of the pyroptosis related gene IL-1β in the BMDMs was higher when the extracellular ATP concentrations increased and lower as the extracellular ATP concentrations dropped (Supplementary Fig. S4). Immunofluorescence co-staining of Cx43 or F4/80 with GSDMD showed that GSDMD distributed mainly around Cx43-positive tubules in the UUO kidneys, and the F4/80- and GSDMD-double positive cells were lower in the C-K UUO kidney, implying that Cx43 depletion in TECs reduced peritubular macrophage pyroptosis (Fig. 6E, F). WB analysis showed that NLRP3, caspase-1, ASC-1 and GSDMD-N were markedly reduced in the C-K UUO kidneys (Supplementary Fig. S5). Therefore, the outflow of ATP from TECs induced the activation of macrophage inflammasomes and triggered macrophage pyroptosis after UUO.

### The expression of Cx43 and GSDMD are associated with renal function decline in human obstructive nephropathy

**Fig. 7:**
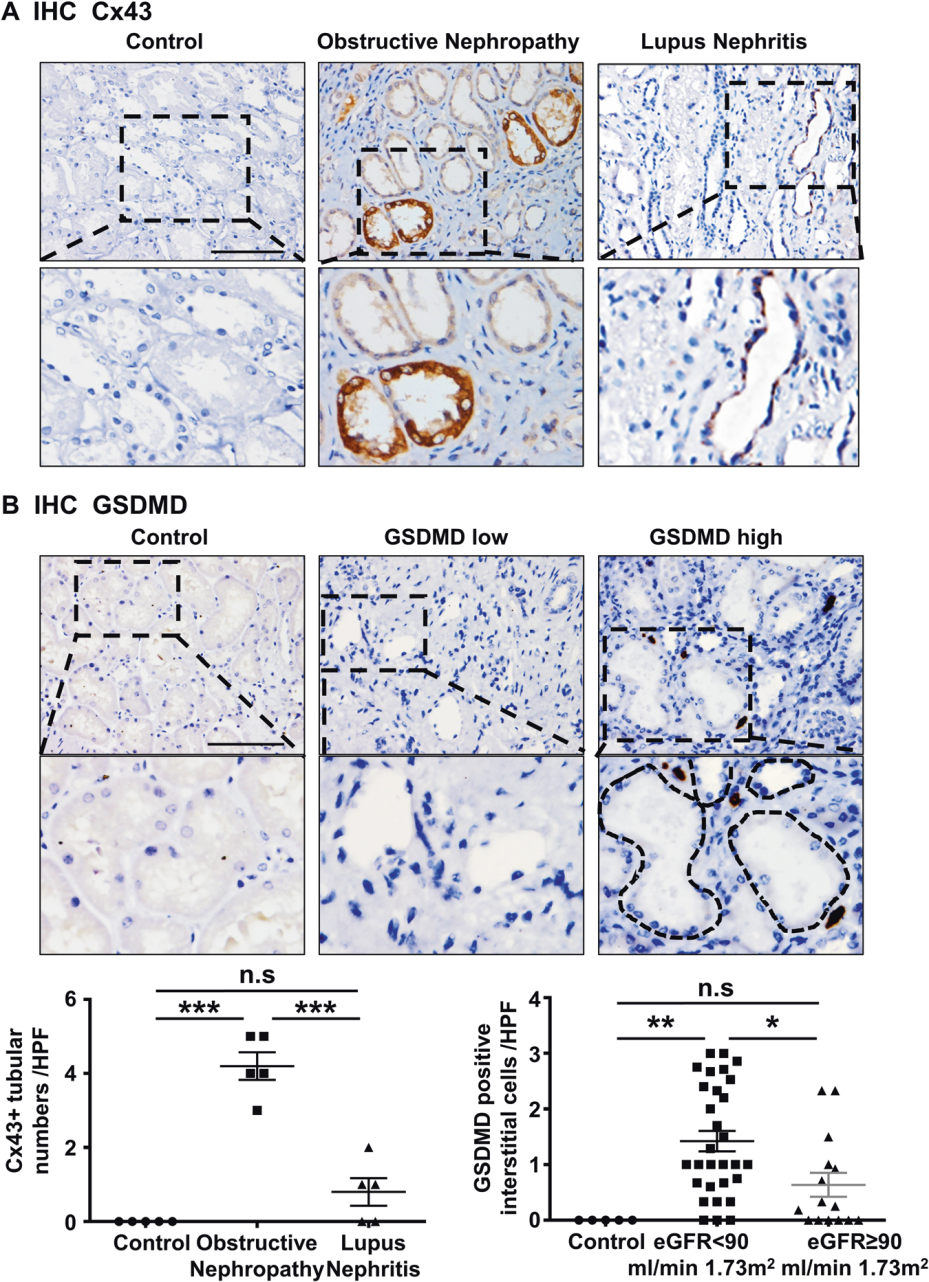
The expression of Cx43 and GSDMD genes was associated with renal function in human obstructive nephropathy. **A** The representative image of Cx43 immunohistochemistry in TECs in human renal obstruction disease and lupus nephritis. **B** The representative image of GSDMD immunohistochemistry in GSDMD low group and GSDMD high group. Data are presented as the mean ± SEM, n.s. no significance, ***P < 0.001; Scale bar: 100 μm.

We detected the expression of Cx43 and GSDMD in human kidney diseases. Immunohistochemical staining showed greater expression of tubular Cx43 in renal biopsy specimens from individuals diagnosed with obstructive nephropathy and lupus nephritis (Fig. 7A). GSDMD was observed to be expressed in renal interstitial cells with obstructive nephropathy. Neither Cx43 nor GSDMD were found in normal kidney specimens. Further, we divided individuals into two groups: those with an eGFR <90 ml/min/1.73 m^2^ and those with an eGFR ≥90 ml/min/1.73 m^2^ group. Peritubular GSDMD high cell was defined as the number of GSDMD-positive cells ≥50th percentiles. Individuals in the eGFR <90 ml/min 1.73 m^2^ group showed higher levels of Cx43 (*P* < 0.015) and GSDMD (*P* < 0.041) at the time of biopsy than the other group (Fig. 7B). Univariate and multivariate binary logistic regression indicated that appearance of Cx43 in TECs and GSDMD in interstitial cells were independent risk factors for the decline of renal function in subjects with obstructive nephropathy (Tables 1, 2).

**Table 1:** Obstructive nephropathy patients in the Cx43 positive group and GSDMD high group showed a decline of kidney function.

Variables	All (*n* = 48)	eGFR ≥ 90 ml/min 1.73 m^2^ (*n* = 16)	eGFR < 90 ml/min 1.73 m^2^ (*n* = 32)	*P*
Gender male, *n*%	23/48 (47.9%)	5/23 (21.7%)	18/23 (78.3%)	0.102^c^
Age, years	48.12 ± 15.11	40.63 ± 13.35	52.75 ± 13.05	**0.004**^**a**^
WBC, 10^9/L	9.84 ± 3.24	10.16 ± 3.49	9.71 ± 3.21	0.658^a^
N, 10^9/L	7.90 ± 3.27	8.15 ± 3.55	7.84 ± 3.18	0.759^a^
L, 10^9/L	1.17 (0.74-1.53)	1.01 (0.70-1.77)	1.21 (0.7-1.52)	0.702^b^
M, 10^9/L	0.63 (0.5-0.8)	0.68 (0.59-0.87)	0.57 (0.41-0.78)	0.137^b^
Hb, g/L	121.44 ± 16.02	117.31 ± 17.91	123.53 ± 14.45	0.201^a^
Platelets, 10^9/L	191 (157-229)	195.5 (152.75-258.5)	189 (157-220.75)	0.519^b^
Na^+^, mmol/L	139.3 ± 2.60	138.53 ± 2.24	139.71 ± 2.75	0.144^a^
K^+^, mmol/L	4.06 ± 0.39	3.96 ± 0.27	4.12 ± 0.44	0.186^a^
Cl^-^, mmol/L	103.84 ± 2.98	103.53 ± 3.37	103.97 ± 2.86	0.642^a^
Ca^2+^, mmol/L	2.18 ± 0.13	2.21 ± 0.11	2.16 ± 0.13	0.227^a^
P, mmol/L	1.05 ± 0.23	1.09 ± 0.19	1.03 ± 0.25	0.507^a^
ALT, U/L	11 (8–21)	10 (6–16)	12 (9-23.50)	0.130^b^
AST, U/L	16 (13.5-21)	15 (12.5-16)	18.5 (15-21.5)	**0.023**^**b**^
TP, g/L	62.87 ± 5.81	61.63 ± 4.52	63.09 ± 6.02	0.396^a^
ALB, g/L	36.54 ± 3.90	35.89 ± 3.56	36.53 ± 3.69	0.569^a^
GLB, g/L	26.40 ± 3.41	25.96 ± 2.50	26.56 ± 3.83	0.573^a^
Chol, mmol/L	3.54 (3.11-4.12)	3.96 (3.33-4.47)	3.46 (3.08-3.96)	0.068^b^
UA, μmol/L	302.20 ± 82.357	273.73 ± 76.20	316.25 ± 84.08	0.096^a^
Proteinuria positive, *n*%	13/48 (27%)	3/13 (23.1%)	10/13 (76.9%)	0.358^c^
Hematuria positive, *n*%	12/48 (25%)	4/12 (33.3%)	8/12 (66.7%)	1.000^c^
pyuria positive, *n*%	8/48 (16.7%)	1/8 (12.5%)	7/8 (87.5%)	0.171^c^
Cx43 positive, *n*%	37/48 (77.1%)	9/37 (24.3%)	28/37 (75.7%)	**0.015**^**c**^
GSDMD high, *n*%	25/48 (52.1%)	5/25 (20%)	20/25 (80%)	**0.041**^**c**^

The bold values indicate *P* < 0.05.

Data are presented as mean ± SD or median (25–75th percentiles) or a percentage.

Peritubular GSDMD high cell was defined as the number of GSDMD positive cells ≥50th percentiles.

*WBC* White blood cell, *N* Neutrophils, *L* Lymphocytes, *M* Monocyte, *TP* Total protein, *ALB* Albumin, *GLB* Globulin, *Chol* Cholesterol, *UA* Uric acid.

^a^t-test, ^b^Mann–Whitney *U* test, ^c^Pearson’s chi-squared test.

**Table 2:** Risk factors associated with eGFR <90 ml/min/1.73 m^2^ during follow-up periods.

Variables	Univariate analysis			Multivariate analysis		
	OR	95%CI	*P*	OR	95%CI	*P*
Gender male	2.829	(0.797–10.042)	0.108			
Age, years	1.07	(1.017–1.126)	**0.009**	1.104	(1.027–1.186)	**0.007**
Cx43 in TECs (positive vs negative) (*n* = 48)	5.444	(1.290–22.976)	**0.021**	10.388	(1.439–74.997)	**0.020**
GSDMD in interstitial cells (high vs low)^a^ (*n* = 48)	3.667	(1.023–13.143)	**0.046**	6.929	(1.189–40.374)	**0.031**

The bold values indicate *P* < 0.05.

^a^GSDMD (GSDMD high was defined as the number of GSDMD positive cells ≥50th percentiles, 1, GSDMD positive cells ≥50th percentiles; 0, GSDMD positive cells <50th percentiles).

### Pyroptotic macrophage-derived CXCL10 aggravates the progression of UUO-induced renal fibrosis

**Fig. 8:**
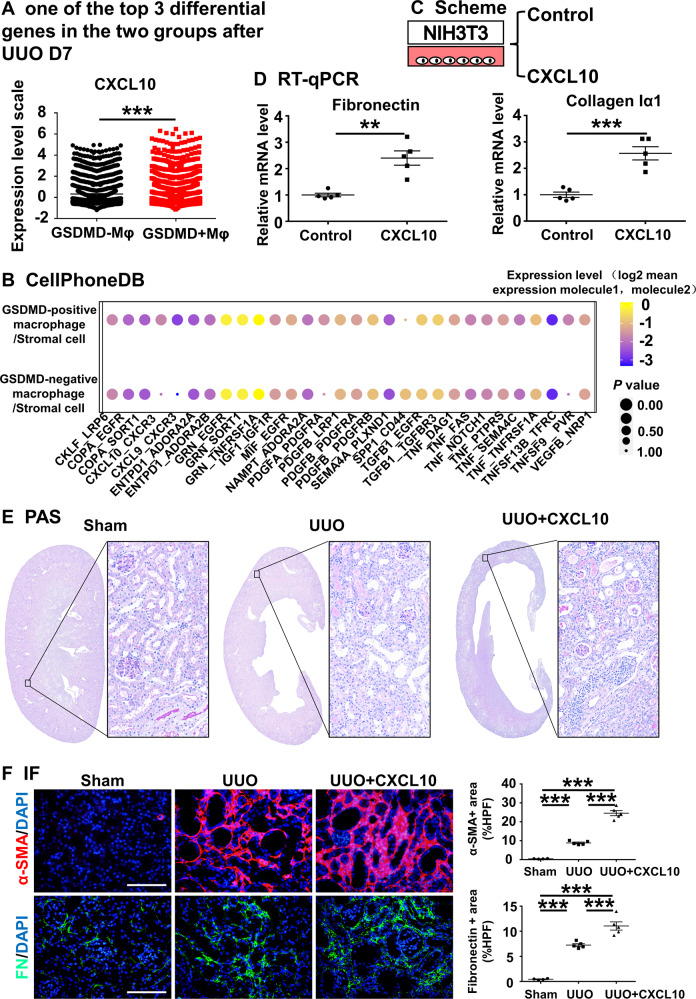
CXCL10 can aggravate the progression of obstructive nephropathy. **A** One of the top 3 differential genes between the GSDMD positive macrophage and GSDMD-negative macrophage was CXCL10 by scRNA-seq. **B** Bubble heatmap showing the mean attraction strength for selected ligand-receptor pairs between the GSDMD-positive/negative macrophage with Stromal cell. Dot size indicates *P*-value generated by permutation test, colored by attraction strength levels. **C** Scheme. **D** Levels of mRNA encoding Fibronectin, collagen I1α by RT-qPCR. **E** Representative overall photo and photomicrographs of PAS staining. **F** Immunofluorescence. Representative photomicrographs for immunofluorescence labeled α-SMA (red) and FN (green). Corresponding graphs indicate the expression of α-SMA (red) and FN (green). Data are presented as the mean ± SEM, *n* = 5/group, **P < 0.01, ***P < 0.001; Scale bar: 100 μm.

To explore the mechanism by which macrophage pyroptosis induces renal fibrosis, we divided macrophages into two groups: the GSDMD-negative and the GSDMD-positive groups, and analyzed them by scRNA-seq. The fibrosis-related chemokine CXCL10 ranked among the top three of differential genes (Fig. 8A). It has been reported that CXCL10 plays a key role in wound healing, pulmonary fibrosis, and liver fibrosis [59–61]. During kidney fibrosis, the transdifferentiation of fibroblasts into myofibroblasts is an indispensable process. In order to confirm whether the GSDMD-positive macrophages were more likely to communicate with fibroblast. We calculated the attraction strengths of ligand-receptor pairs in our scRNA-seq dataset by using a simulation analysis similar to previous methods [62, 63]. Of the ligand receptor pairs pertaining to macrophage and stromal cell (including fibroblast), CXCL10-CXCR3 was significantly enriched in GSDMD-positive macrophages compared to GSDMD-negative macrophages in UUO kidneys (Fig. 8B), implicating a potential role of GSDMD-positive macrophage in recruiting or activating fibroblasts. Thus, we cultured NIH3T3 cells with CXCL10 in vitro and found that CXCL10 aggravated fibrosis. This result implies that CXCL10 from GSDMD-positive macrophages participates in the activation of fibroblasts (Fig. 8C,D).

Next, we applied CXCL10 to UUO mice. We found that this treatment resulted in more serious tubular injury and fibrosis compared to the untreated UUO group (Fig. 8E, F). These data demonstrate that macrophage-derived CXCL10 after pyroptosis accelerates UUO-induced renal fibrosis.

## Discussion

The expression and distribution of GJs have been reported to have a significant impact on various diseases [20, 22, 25, 64]. In this study, we demonstrated that Cx43-positive TECs communicate with macrophages through release of ATP and promotion of renal fibrosis, while inhibiting or blocking Cx43 improved renal structure and function.

The expression and distribution of Cx43 has been reported in various inflammatory diseases regulating the progression of inflammatory injury [20, 65–67]. Renal tubular epithelial cell injury is one of the key causes of renal fibrosis. Injured TECs aggravate renal fibrosis by releasing inflammatory factors, chemokines, and exosomes [9]. In addition to exosomes and exocytosis, some metabolites are directly released through specific channels, such as GJ channels. Among these, Cx43 is the most widely expressed and the best studied [25, 26]. Cx43 expression has a strong correlation with myocardial fibrosis, liver fibrosis, and pulmonary fibrosis [32, 68–71], but it has been less studied in renal fibrosis. Heqing Huang et al. demonstrated that the expression of Cx43 prevented the progression of diabetic RIF [34]. However, Chadjichristos et al. determined that the progression of CKD is delayed after Cx43 is systemically blocked [15]. Therefore, the role of Cx43 on the kidney is controversial and worthy of study. In the kidney, Cx43 is distributed on various types of kidney cells [16, 19, 72, 73]. Here, we showed that Cx43 expression is higher on collecting duct cells after renal injuries. To investigate whether Cx43 mediated renal fibrosis, we took advantage of gene editing technology to construct Cx43-KSP mice. We found that knockout of Cx43 on TECs alleviates renal injury and fibrosis. Through transcriptome and metabolomics analysis, we confirmed that Cx43 aggravates renal fibrosis by mediating ATP outflow. It has been demonstrated that extracellular ATP acting as a damage associated molecular pattern (DAMP) in various diseases is sensed by P2X receptors where its activation mainly promotes the formation of inflammasomes and the initiation of pyroptosis [74–76]. Our previous findings showed that pyroptosis after kidney injury mainly occurs in macrophages [77]. By scRNA-seq, we found that the P2X receptor in the injured kidney is mainly distributed on macrophages and participates in macrophage pyroptosis. This result is consistent with previously reported results [57]. We further confirmed here that Cx43 activates renal macrophage pyroptosis by facilitating the release of ATP from neighboring TECs, which in turn binds to the P2X receptor on macrophages.

Previous studies have shown that macrophage pyroptosis mainly promotes inflammation. By using scRNA-seq, we found that CXCL10 is one of the top three differentially expressed genes between GSDMD-positive and GSDMD-negative macrophages after UUO. Previous reports have shown that CXCL10 is strongly positively correlated with the progression of organ fibrosis, such as skin, liver, and lung [59–61]. Our study confirmed that CXCL10 directly activates the proliferation of fibroblasts. Thus, the present study provides new insight into the mechanism of renal fibrosis.

While pharmacological agents that specifically target the hemichannel function of on TECs is not available, by using nanotechnology to embed Cx43 hemichannel blockers into nanomaterials with renal tubular specific targeting it is possible that such a future therapy may be developed. In spite of this current limitation, our study provides important new insight as it provides evidence for the protective effect of targeting tubular Cx43 on renal fibrosis via inhibiting ATP outflow and the resulting macrophage pyroptosis. Together, this insight provides a novel mechanism and a potential therapeutic strategy to prevent renal fibrosis.

## Methods

### Animal model

All mice were kept in well-controlled animal housing facilities with a 12 h day and night cycle, had free access to tap water and pellet food in accordance with the Experimental Animal Ethics Committee of Huazhong University of Science and Technology. Male C57BL/6 mice (8–10 weeks old, weighing 22–25 g, Vital River Laboratory, Beijing, China) were anaesthetized with 1% sodium pentobarbital solution (0.009 ml/g, Sigma, USA) by intraperitoneal injection. The animal genotype was not known when the animal model was constructed and the medicine was administered. Finally, it was judged by the mouse ear mark.

Unilateral ureteral obstruction (UUO) model: The left ureter was ligated with 4-0 silk at 2 points, close to the renal pelvis. Then the muscle layer and skin were closed with 4-0 silk. Sham animal models were subjected to a similar surgical procedure without ligating the left ureter.

Bilateral ischemia-reperfusion injury (BIR) model: both renal pedicles were clamped with an atraumatic vascular clip for 30 min (Roboz Surgical Instrument Co, Germany). The technical success of ischemia-reperfusion was checked by observing the kidney color after clamping and after removing the clamps. The body temperature was controlled at 36.6–37.2 °C by a sensitive rectal probe throughout the procedure (FHC, USA). Then, the muscle layer and skin were closed with 4-0 silk. Sham operations were performed with exposure of the left kidney, but without induction of ischemia.

All animals were housed in the specific pathogenfree laboratory animal center of Huazhong University of science and technology ([2017] IACUC Number: 2471).

### Cx43-KSP gene mice and chemical

Cx43-loxp mice were purchased from Jackson Laboratory, USA. KSP-icreERT mice were developed in cooperation with Beijing Biocytogen company Corporation using CRISPR/Cas9 technology. Cx43-KSP gene mice were generated by crossing Cx43-loxp gene mice and KSP-icreERT mice to deplete the Cx43 gene of renal tubular epithelial cells. In vivo experiment, we used different concentrations of 4-OHT to induce Cx43 knockout and found 10 μM was the best dose. The control mice were for Cx43-loxp or KSP-icreERT mice. A rang doses of 0.01 μg/μl, 0.02 μg/μl, 0.04 μg/μl were injected into the mice with GAP26 for 8 days.

#### In vitro-based model

In vitro, the pTECs from C-K mice were cultured with 4-OHT, an active form of tamoxifen, to knockdown Cx43, we used a rang doses of 0.1 μM, 1 μM, 10 μM, and simultaneously treated with TGF-β (10 ng/ml) for 12 h.

Cellular pyroptosis model: By stimulating the BMDM cells with 5 mM ATP alone for 8 h, or with 500 ng/ml LPS alone for 4 h and then with 5 mM ATP alone for 8 h [78].

### Histology and immunofluorescence

Periodic acid-schiff (PAS) staining was used to evaluate kidney pathological injury, and Sirius Red (SR) staining were carried out to estimate the extent of tubular interstitial fibrosis.

Immunofluorescence (IF) renal sections were dewaxed in a constant temperature oven and subjected to heat antigen retrieval in a microwave oven. The nonspecific antigens were blocked with serum for 30 min at room temperature. The slides were then incubated with specific primary antibodies against Cx43 (1:100, Sigma, USA), KIM-1 (1:1000, R&D system, USA), LTL (1:50, Vector Laboratories, USA), NCC (1:100, Millipore, USA), DBA (1:300, Vector Laboratories, USA), KSP (1:100, Proteintech, China), α-SMA (1:100, Abcam, UK), Fibronectin (1:100, R&D system, USA), GSDMD (1:300, Santa cruz, USA) and F4/80 (1:100, Abcam, UK) at 4 °C for 24 h. Then labeling fluorescent secondary antibodies for IF. Nuclei were stained with DAPI. Staining was carefully quantified in each slide by capturing ten randomly visions in a blind manner by two experienced renal pathologists and the data was analyzed by Image Pro Plus software (Media Cybernetics, Rockville, MD, USA).

### Western blotting

Renal tissues were lysed in RIPA lysis buffer (Promoter, Wuhan, China) containing protease inhibitors (Promoter, Wuhan, China). Equal amounts of proteins (40 μg) were loaded and separated by SDS-PAGE. The gel was transferred onto PVDF membranes (Millipore, Billerica, MA, USA). The membranes were blocked with 5% skimmed milk in TBST for 1 h at 37 °C and were then incubated with primary antibodies against Cx43 (1:1000, sigma, USA), GAPDH (1:5000, Abclonal, China), NLRP3 (1:1000, CST, USA), ASC-1 (1:1000, CST, USA), Caspase-1 (1:1000, CST, USA) and GSDMD (1:1000, Santa cruz, USA) at 4 °C overnight. The PVDF membranes were incubated with HRP-conjugated secondary antibodies for 1 h at 37 °C and were visualized by enhanced chemiluminescence (ECL, Biosharp, China). The signal intensity of the targeted band was quantified using Image J (NIH, USA).

### Quantitative real time-PCR

Total RNA was extracted from renal tissues using Trizol reagent according to the manufacturer’s instructions (Invitrogen, USA). Reverse transcribed into first strand cDNA using the reverse transcription system (Vazyme, Nanjing, China). Quantitative PCR was conducted using the SYBR mastermix (Vazyme, Nanjing, China) on the ABI Step-One. Relative mRNA expression levels were calculated using the 2^−ΔΔCt^ method and normalized to the expression levels of GAPDH. The primers used are listed in the table below.

**Table:** 

Gene name	Forward	Reverse
GAPDH	5′-TGACCTCAACTACATGGTCTACA-3′	5′-CTTCCCATTCTCGGCCTTG-3′
Cx43	5′-ACAAGGTCCAAGCCTACTCCA-3′	5′-CCGGGTTGTTGAGTGTTACAG-3′
α-SMA	5′-CCCAGACATCAGGGAGTAATGG-3′	5′-TCTATCGGATACTTCAGCGTCA-3′
Fibronectin	5′-GCTCAGCAAATCGTGCAGC-3′	5′-CTAGGTAGGTCCGTTCCCACT-3′
PDGFR-β	5′-AGGAGTGATACCAGCTTTAGTCC-3′	5′-CCGAGCAGGTCAGAACAAAGG-3′
Collagen Ia1	5′-GCTCCTCTTAGGGGCCACT-3′	5′-CCACGTCTCACCATTGGGG-3′
IL-1β	5′-GTGGCTGTGGAGAAGCTGTG-3′	5′-GAAGGTCCACGGGAAAGACAC-3′

Relative mRNA expression levels were quantified according to the 2^−ΔΔCt^ method and were normalized to the expression levels of GAPDH.

### Flow cytometry

Mouse kidney tissue was chopped and digested with Collagenase-IV (Promoter, Wuhan, China) at 37 °C for 1 h. The suspension was filtered through a 200 mesh and a 400 mesh filter cloth to generate a Single-cell suspension after lysis of red blood cells (BD, USA). The cells were then incubated with the following fluorescent antibodies for 30 min shielded from light at room temperature: FITC-conjugated anti-CD45 (Biolegend, USA), APC-conjugated anti-KSP (Novus, USA), PE-conjugated anti-Cx43 (Santa Cruz, USA), Zombie Violet™ Fixable Viability Kit (Biolegend, USA). The cells were sorted using a Beckman CytoFLEX and the data were analysed using the CytoExpert for DxFLEX software. We added 10 µL of precision-count beads to each sample before sorting cells. The absolute cell count was calculated by using the following formula: Absolute Cell Count = $\left( {\frac{{{{{\mathrm{Cells}}}}}}{{{{{\hat{\mathrm A}}}}\,\mu {{{\mathrm{L}}}}}}} \right) = \frac{{{{{\mathrm{Cell}}}}\,{{{\mathrm{Count}}}}}}{{{{{\mathrm{Precision}}}} - {{{\mathrm{Count}}}}\,{{{\mathrm{Bead}}}}\,{{{\mathrm{Count}}}}}}$ x Precision-Count Bead Concentration $( {\frac{{{{{\mathrm{Beads}}}}}}{{{{{\hat{\mathrm A}}}}\,\mu {{{\mathrm{L}}}}}}} )$.

### Transcriptome sequencing and bioinformatics analysis

Total RNA was obtained from TECs using Trizol reagent (Invitrogen, USA). The total RNAs were subjected to cDNA synthesis, fragmentation, adapter ligation, and amplification. Sequencing was performed on Illumina HiSeq platform. The sequencing reads were further processed with determination of quality using the SOAPnuke tool. We mapped clean reads to mouse mm9 genome using HISAT (Hierarchical Indexing for Spliced Alignment of Transcripts). The fragments per kilobase million (FPKM) and differential expression genes (DEGs) were obtained using RSEM and DEGseq software, respectively (Fold Change ≥2 and Adjusted *P* value ≤ 0.001). R package was used for generation of GO and KEGG analysis.

### Metabolomics

Metabolomics project data analysis based on Ultra High Performance Liquid Tandem Chromatography Quadrupole Time of Flight Mass Spectrometry, UHPLC-QTOFMS is mainly divided into three parts: basic data analysis, advanced data analysis and optional data analysis. Basic data analysis is to carry out univariate statistical analysis and multivariate statistical analysis of the qualitative and quantitative results of the metabolome, and to screen the metabolites with significant differences; optional data analysis is based on the basic data analysis to conduct significant differences in metabolites Series of bioinformatics analysis. The R packages include bubble plot: ggplot2 ggrepel, heatmap: pheatmap, PCA: ggplot2, network: igraph ggraph.

Commercial databases including KEGG (http://www.genome.jp/kegg/) and MetaboAnalyst (http://www.metaboanalyst.ca/) were used for pathway enrichment analysis.

### 10× Genomics

Single cells in nanoliter-scale oil droplets by Chromium Controller and to generate Gel Bead-In-EMulsions (GEMs). Full length cDNA libraries were prepared by incubation of GEMs in a thermocycler machine. GEMs containing cDNAs were crushed and all single-cell cDNA libraries were collected together, cleaned using DynaBeads MyOne Silane beads (PN 37002D; Fisher). The final constructed single-cell libraries were sequenced by Illumina Novaseq6000 machine with total reads per cell targeted, for a minimum of 50,000.

### Analysis of single-cell RNA-seq (scRNA-seq) data

#### Dimension reduction

Principal component analysis was then performed on significantly variable genes. The variable genes were selected based on dispersion of binned variance to mean expression ratios using Find Variable Genes function of Seurat package and removed unwanted cells. Then the appropriate number principal components were selected as input for clustering. We performed dimension reduction using gene expression data for a subset of variable genes.

#### Determining cluster markers

Differential gene expression testing was performed using the Find Markers function in Seurat with parameter “test.use = wilcox” by default and the Benjamini-Hochberg methods was used to estimate the false discovery rate (FDR). DEGs were filtered using a minimum natural log (fold change) of 0.25 and a maximum FDR value of 0.05.

#### Basic information of clinical obstructive nephropathy patients collected from Tongji Hospital

A total of 48 patients were included in the study who were diagnosis obstructive nephropathy by ultrasonography and relieve the obstruction through surgery. We obtained the related data from electronic medical records and the paraffin section of obstruction nephrology patients from ward of department of urology, Tongji Hospital of Tongji Medical college, Huazhong University of Science and Technology in Wuhan, China. The investigations were conducted in accordance with the principles of the Declaration of Helsinki and were approved by the Institutional Review Board at Tongji Medical College, Huazhong University of Science and Technology (TJ-IRB20210815) and obtaining the informed consent of the patient.

### Statistical analysis

For single cell RNA-seq, the statistical analysis was conducted using the R machine language, including the graphs made by R packages used: Seurat/clusterProfiler/tidyverse. We identify a PC threshold by calculating where the principal components start to elbow by taking the larger value of: (a) The point where the principal components only contribute 5% of standard deviation and the principal components cumulatively contribute 90% of the standard deviation; (b) The point where the percent change in variation between the consequtive PCs is less than 0.1%. Here the pc number is 40. Single cell RNAseq methodology: alignment: STAR software built-in CellRaner software; mitochondrial DNA thresholds least than 20%; NormalizeData: By default, the raw counts are normalized using global-scaling normalization by performing the following: (a) normalizing the gene expression measurements for each cell by the total expression; (b) multiplying this by a scale factor (10,000 by default); (c) log-transforming the result.

Data were prepared using GraphPad Prism software version 6.0 or IBM SPSS Statistics 23. Data conforming to normal distribution were presented as mean ± SD, or median and quartiles for non-normal distribution. Rate comparisons were performed by chi-squared test. *t*-Test, Wilcoxon rank-sum test, Wilcoxon signed-rank test, or Kruskal–Wallis test were used to compare means across groups according to the number of group and distribution of variable. Preliminary univariate binary logistic regression was used to select and estimate the association between Cx43, GSDMD, hematuria, or eGFR and variables that were clinically relevant on grounds of professional knowledge and Stepwise multivariate binary logistic regression was used to select the predictors. Specifically, variables with *P* < 0.05 in the univariate analysis were entered into multivariate analysis to select the predictors (inclusion criterion was *P* < 0.05 and exclusion criterion was *P* ≥ 0.05). Results are presented as odds ratios (ORs) with 95% confidence intervals (95% CIs) and *P* values. All the statistical analyses were two-tailed. n.s. no significance, *P < 0.05, **P < 0.01, ***P < 0.001.

## Supplementary information

Obstructive nephropathy patients in the Cx43 positive group and GSDMD high group showed a decline of kidney function

Risk factors associated with eGFR < 90 ml/min/1.73 m^2^ during follow-up periods

Time-related comparison after UUO and BIR.

The Cx43 gene knockout mice were constructed

P2X7 receptor modulate renal injury and RIF

ATP outflow from renal tubular epithelial cells directly reduced BMDM pyroptosis in vitro

Knockout Cx43 gene alleviated kidney pyroptosis

Original Data File

Reproducibility Checklist

## Data Availability

The raw data acquired for this study are available from the corresponding author on reasonable request.
