# Supplementary material for: Blocking connexin 43 and its promotion of ATP release from renal tubular epithelial cells ameliorates renal fibrosis
Source: Cell Death Dis. 2022 May 31;13(5):511. doi: 10.1038/s41419-022-04910-w (PMC9156700; doi:10.1038/s41419-022-04910-w)
Supplement: Supplementary file 1 — Obstructive nephropathy patients in the Cx43 positive group and GSDMD high group showed a decline of kidney function [file 41419_2022_4910_MOESM1_ESM.docx]

**Table 1. Obstructive nephropathy patients in the Cx43 positive group and GSDMD^high^ group showed a decline of kidney function**

| **Variables** | **All (n=48)** | **eGFR ≥ 90 ml/min 1.73 m^2^ (n=16)** | **eGFR < 90 ml/min 1.73 m^2^ (n=32)** | ***P*** |
| --- | --- | --- | --- | --- |
| **Gender male, n%** | 23/48 (47.9%) | 5/23 (21.7%) | 18/23 (78.3%) | 0.102^c^ |
| **Age, years** | 48.12±15.11 | 40.63±13.35 | 52.75±13.05 | **0.004^a^** |
| **White blood cell, 10^9/L** | 9.84±3.24 | 10.16±3.49 | 9.71±3.21 | 0.658^a^ |
| **N, 10^9/L** | 7.90±3.27 | 8.15±3.55 | 7.84±3.18 | 0.759^a^ |
| **L, 10^9/L** | 1.17 (0.74-1.53) | 1.01 (0.70-1.77) | 1.21 (0.7-1.52) | 0.702^b^ |
| **monocyte, 10^9/L** | 0.63 (0.5-0.8) | 0.68 (0.59-0.87) | 0.57 (0.41-0.78) | 0.137^b^ |
| **Hb, g/L** | 121.44±16.02 | 117.31±17.91 | 123.53±14.45 | 0.201^a^ |
| **Platelets, 10^9/L** | 191 (157-229) | 195.5 (152.75-258.5) | 189 (157-220.75) | 0.519^b^ |
| **Na, mmol/L** | 139.3±2.60 | 138.53±2.24 | 139.71±2.75 | 0.144^a^ |
| **K, mmol/L** | 4.06±0.39 | 3.96±0.27 | 4.12±0.44 | 0.186^a^ |
| **Cl, mmol/L** | 103.84±2.98 | 103.53±3.37 | 103.97±2.86 | 0.642^a^ |
| **Ca, mmol/L** | 2.18±0.13 | 2.21±0.11 | 2.16±0.13 | 0.227^a^ |
| **P, mmol/L** | 1.05±0.23 | 1.09±0.19 | 1.03±0.25 | 0.507^a^ |
| **ALT, U/L** | 11 (8-21) | 10 (6-16) | 12 (9-23.50) | 0.130^b^ |
| **AST, U/L** | 16 (13.5-21) | 15 (12.5-16) | 18.5 (15-21.5) | **0.023^b^** |
| **TP, g/L** | 62.87±5.81 | 61.63±4.52 | 63.09±6.02 | 0.396^a^ |
| **ALB, g/L** | 36.54±3.90 | 35.89±3.56 | 36.53±3.69 | 0.569^a^ |
| **GLB, g/L** | 26.40±3.41 | 25.96±2.50 | 26.56±3.83 | 0.573^a^ |
| **Chol, mmol/L** | 3.54 (3.11-4.12) | 3.96 (3.33-4.47) | 3.46 (3.08-3.96) | 0.068^b^ |
| **UA, μmol/L** | 302.20±82.357 | 273.73±76.20 | 316.25±84.08 | 0.096^a^ |
| **Proteinuria positive, n%** | 13/48 (27%) | 3/13 (23.1%) | 10/13 (76.9%) | 0.358^c^ |
| **Hematuria positive, n%** | 12/48 (25%) | 4/12 (33.3%) | 8/12 (66.7%) | 1.000^c^ |
| **pyuria positive, n%** | 8/48 (16.7%) | 1/8 (12.5%) | 7/8 (87.5%) | 0.171^c^ |
| **Cx43 positive, n%**  **GSDMD high, n%** | 37/48 (77.1%)  25/48 (52.1%) | 9/37 (24.3%)  5/25 (20%) | 28/37 (75.7%)  20/25 (80%) | **0.015^c^**  **0.041^c^** |

**a: t-test, b: Mann Whitney U test, c: Pearson's chi-squared test**

**Data are presented as mean ± SD or median (25th–75th percentiles) or a percentage.**

**Peritubular GSDMD high cell was defined as the number of GSDMD positive cells ≥ 50th percentiles**
