# Supplementary material for: Blocking connexin 43 and its promotion of ATP release from renal tubular epithelial cells ameliorates renal fibrosis
Source: Cell Death Dis. 2022 May 31;13(5):511. doi: 10.1038/s41419-022-04910-w (PMC9156700; doi:10.1038/s41419-022-04910-w)
Supplement: Supplementary file 2 — Risk factors associated with eGFR < 90 ml/min/1.73 m2 during follow-up periods [file 41419_2022_4910_MOESM2_ESM.docx]

**Table 2. Risk factors associated with eGFR < 90 ml/min/1.73 m^2^ during follow-up periods**

| **Variables** | **Univariate analysis** | | | **Multivariate analysis** | | |
| --- | --- | --- | --- | --- | --- | --- |
|  | **OR** | **95%CI** | ***P*** | **OR** | **95%CI** | ***P*** |
| **Gender male** | 2.829 | (0.797-10.042) | 0.108 |  |  |  |
| **Age, years** | 1.07 | (1.017-1.126) | **0.009** | 1.104 | (1.027-1.186) | **0.007** |
| **Cx43 in TECs (positive vs negative) (n=48)** | 5.444 | (1.290-22.976) | **0.021** | 10.388 | (1.439-74.997) | **0.020** |
| **GSDMD in interstitial cells (high vs low)^a^ (n=48)** | 3.667 | (1.023-13.143) | **0.046** | 6.929 | (1.189-40.374) | **0.031** |

**a. GSDMD (GSDMD high was defined as the number of GSDMD positive cells ≥ 50th percentiles, 1, GSDMD positive cells ≥ 50th percentiles; 0, GSDMD positive cells < 50th percentiles)**
