# Supplementary figures and images for: Blocking connexin 43 and its promotion of ATP release from renal tubular epithelial cells ameliorates renal fibrosis
Source: Cell Death Dis. 2022 May 31;13(5):511. doi: 10.1038/s41419-022-04910-w (PMC9156700; doi:10.1038/s41419-022-04910-w)

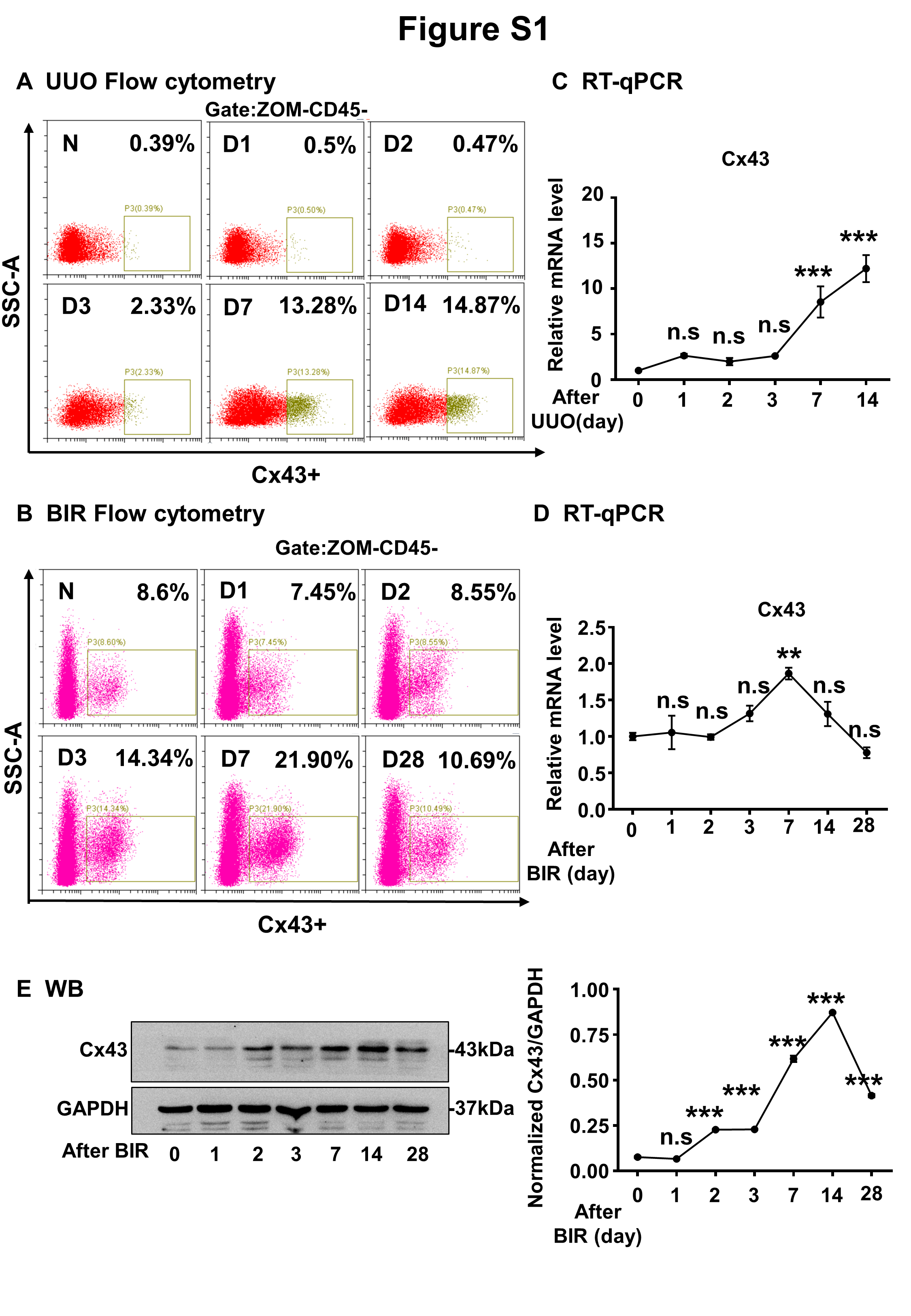

Supplement: Supplementary file 3 — Time-related comparison after UUO and BIR. [file 41419_2022_4910_MOESM3_ESM.tif]

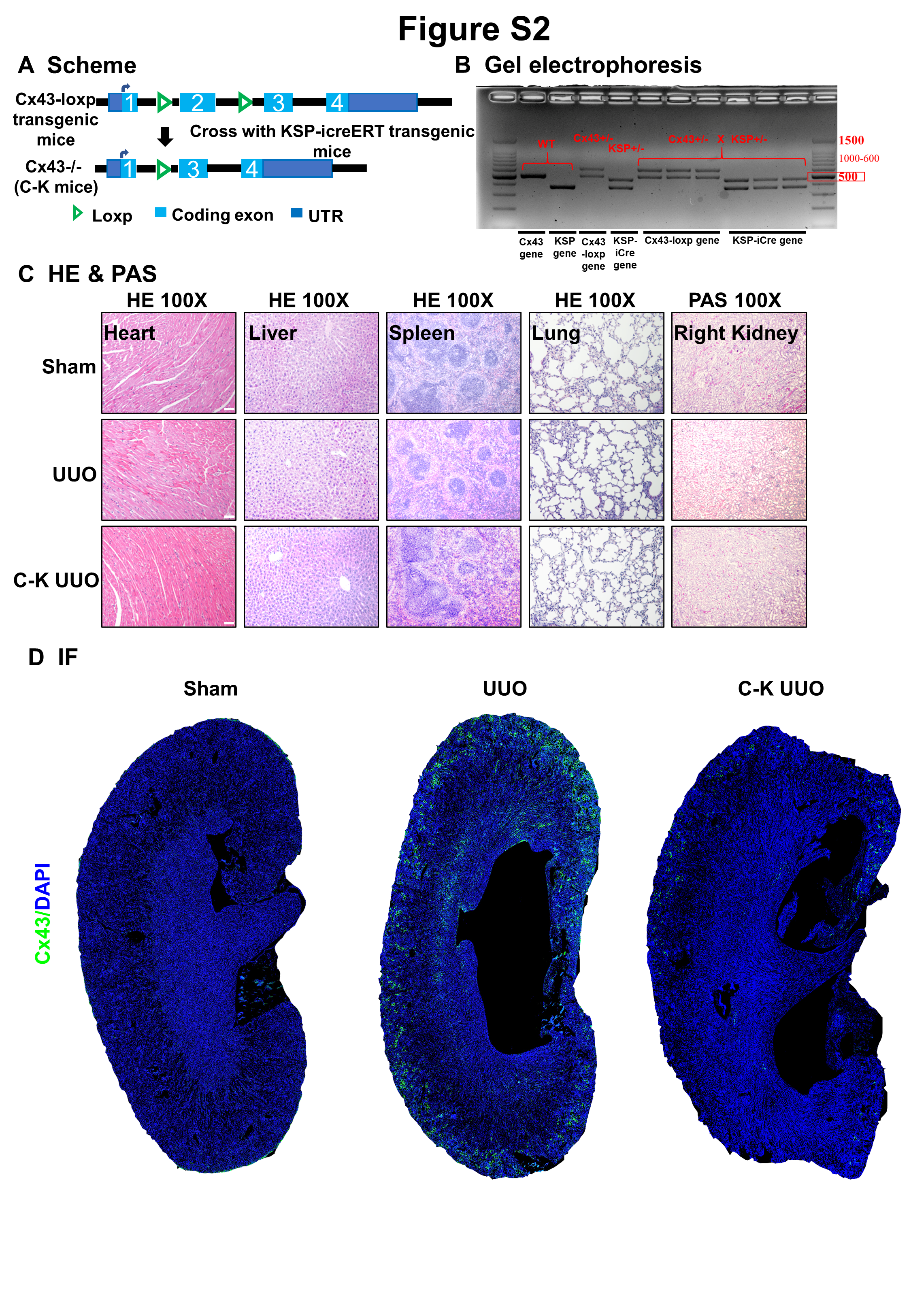

Supplement: Supplementary file 4 — The Cx43 gene knockout mice were constructed [file 41419_2022_4910_MOESM4_ESM.tif]

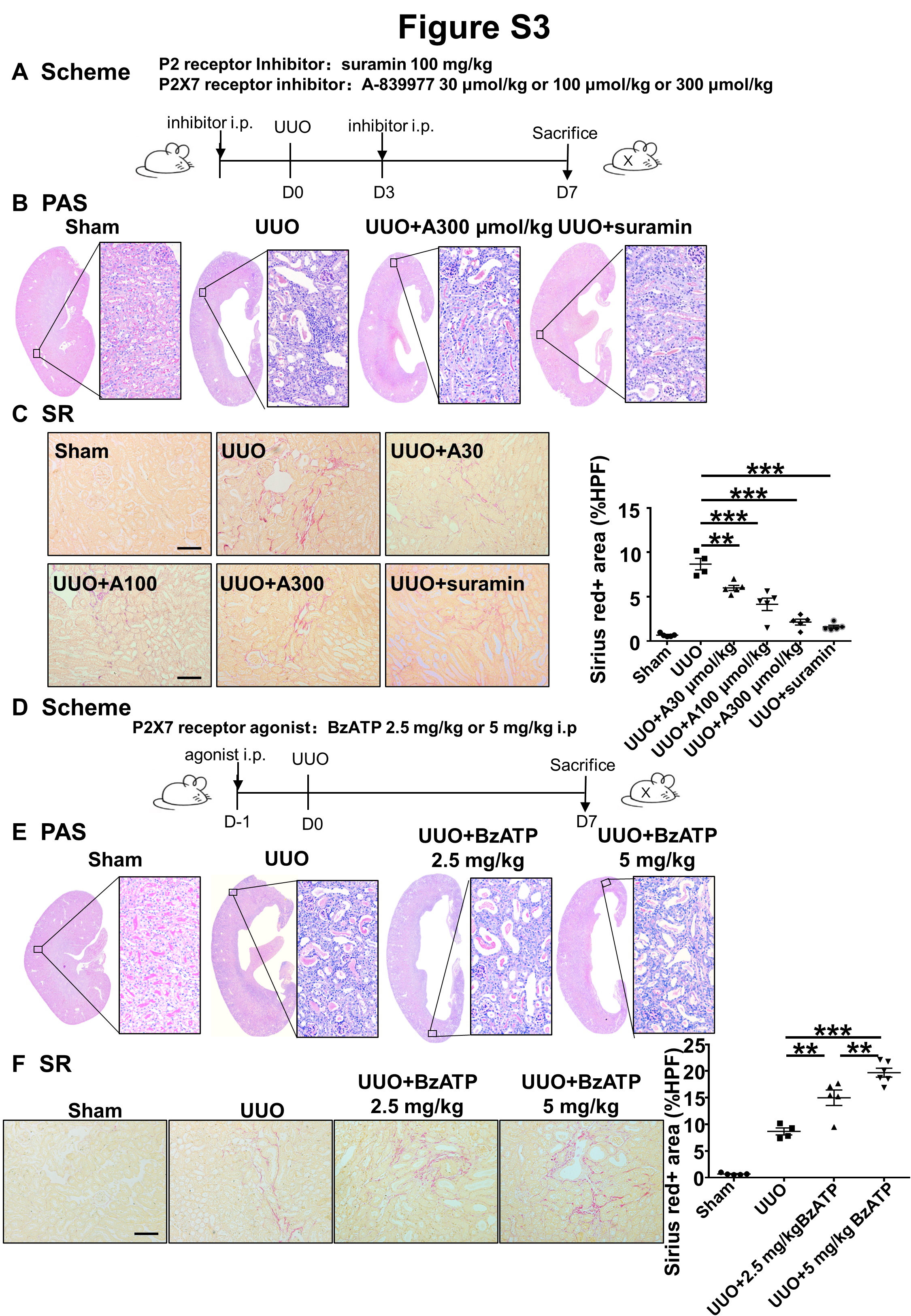

Supplement: Supplementary file 5 — P2X7 receptor modulate renal injury and RIF [file 41419_2022_4910_MOESM5_ESM.tif]

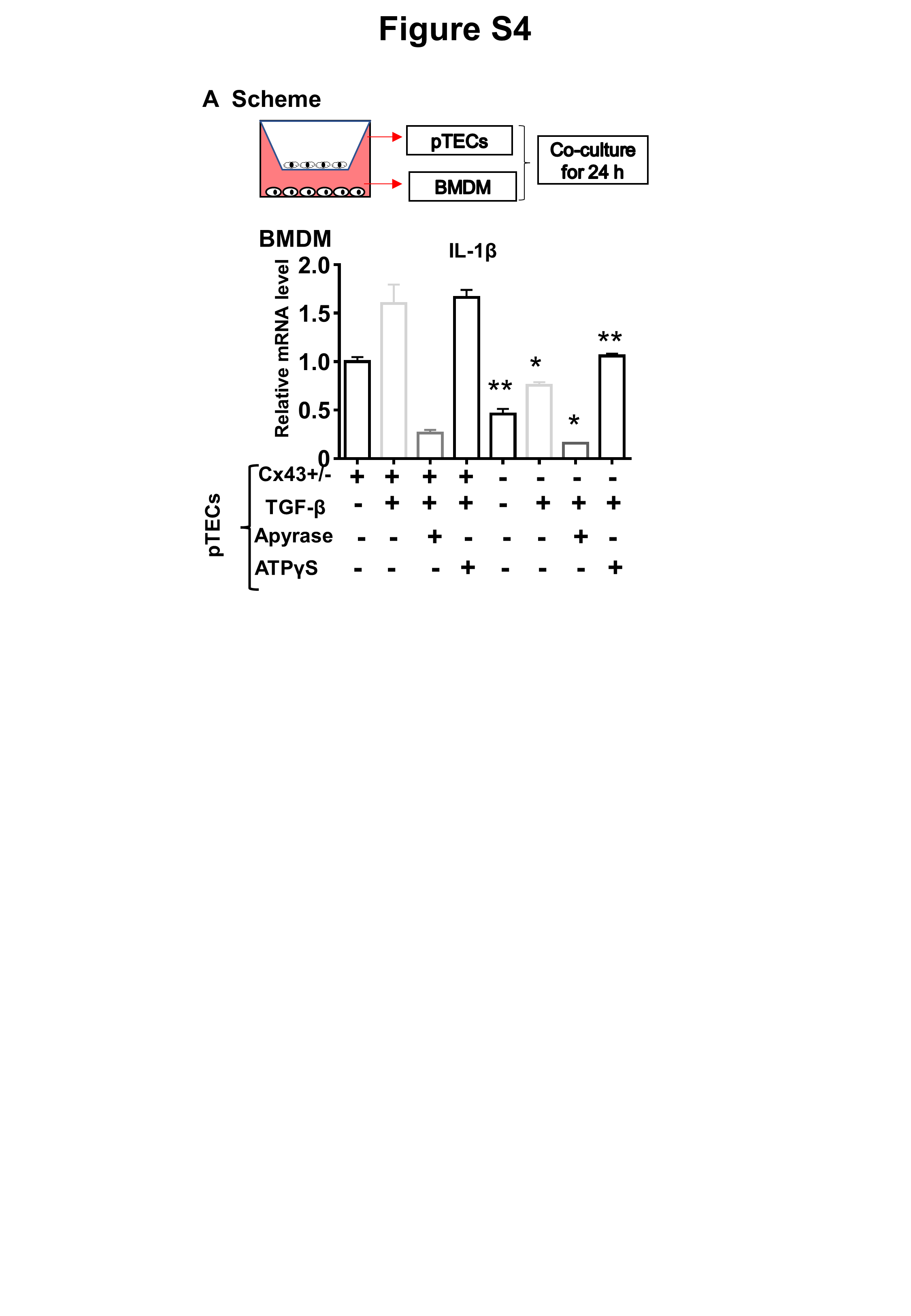

Supplement: Supplementary file 6 — ATP outflow from renal tubular epithelial cells directly reduced BMDM pyroptosis in vitro [file 41419_2022_4910_MOESM6_ESM.tif]

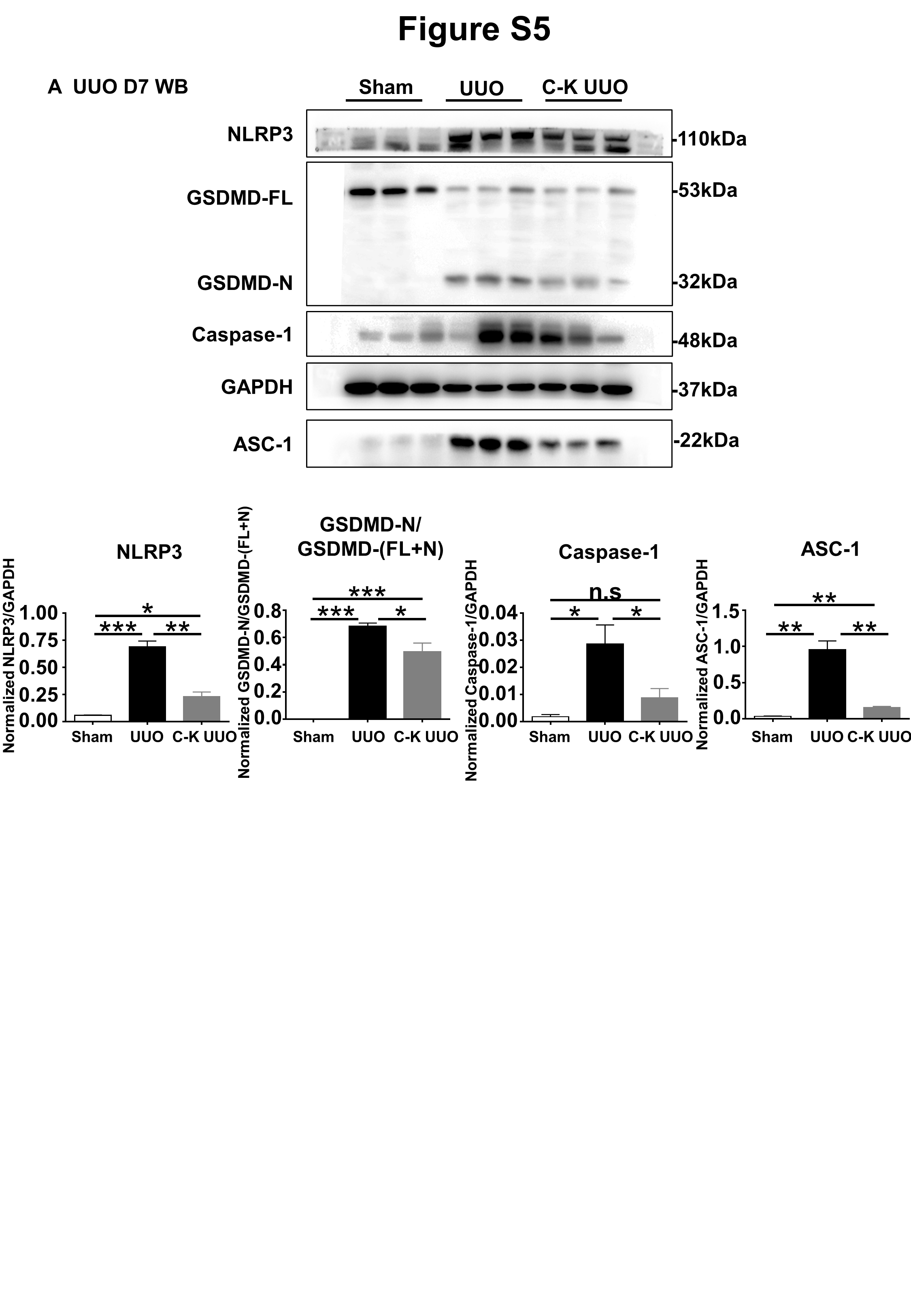

Supplement: Supplementary file 7 — Knockout Cx43 gene alleviated kidney pyroptosis [file 41419_2022_4910_MOESM7_ESM.tif]

WB uncropped

Figure 6C

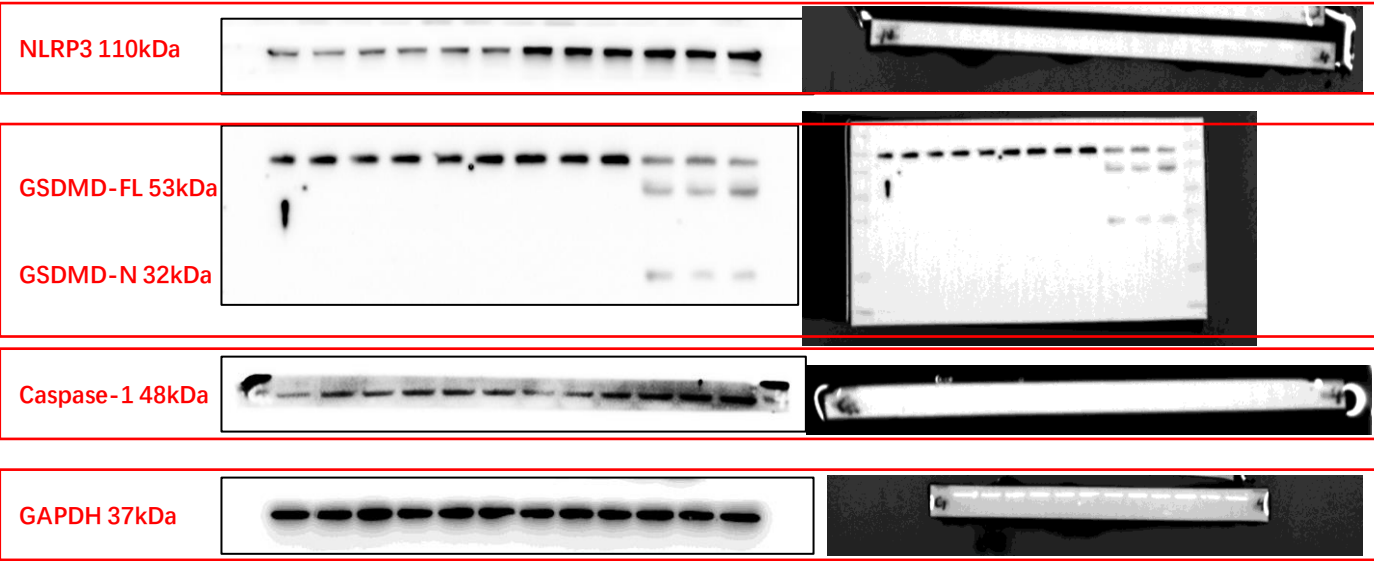

Figure S1E

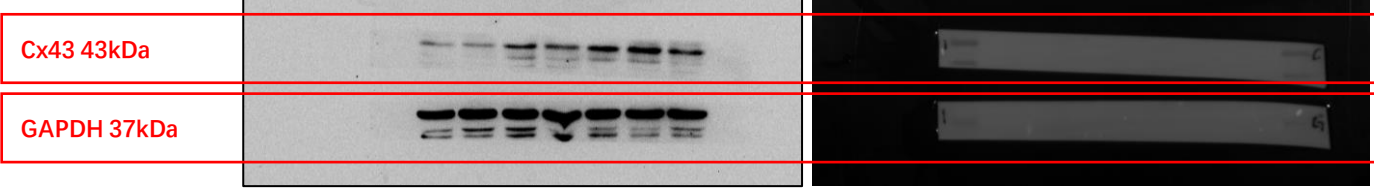

Figure S5A

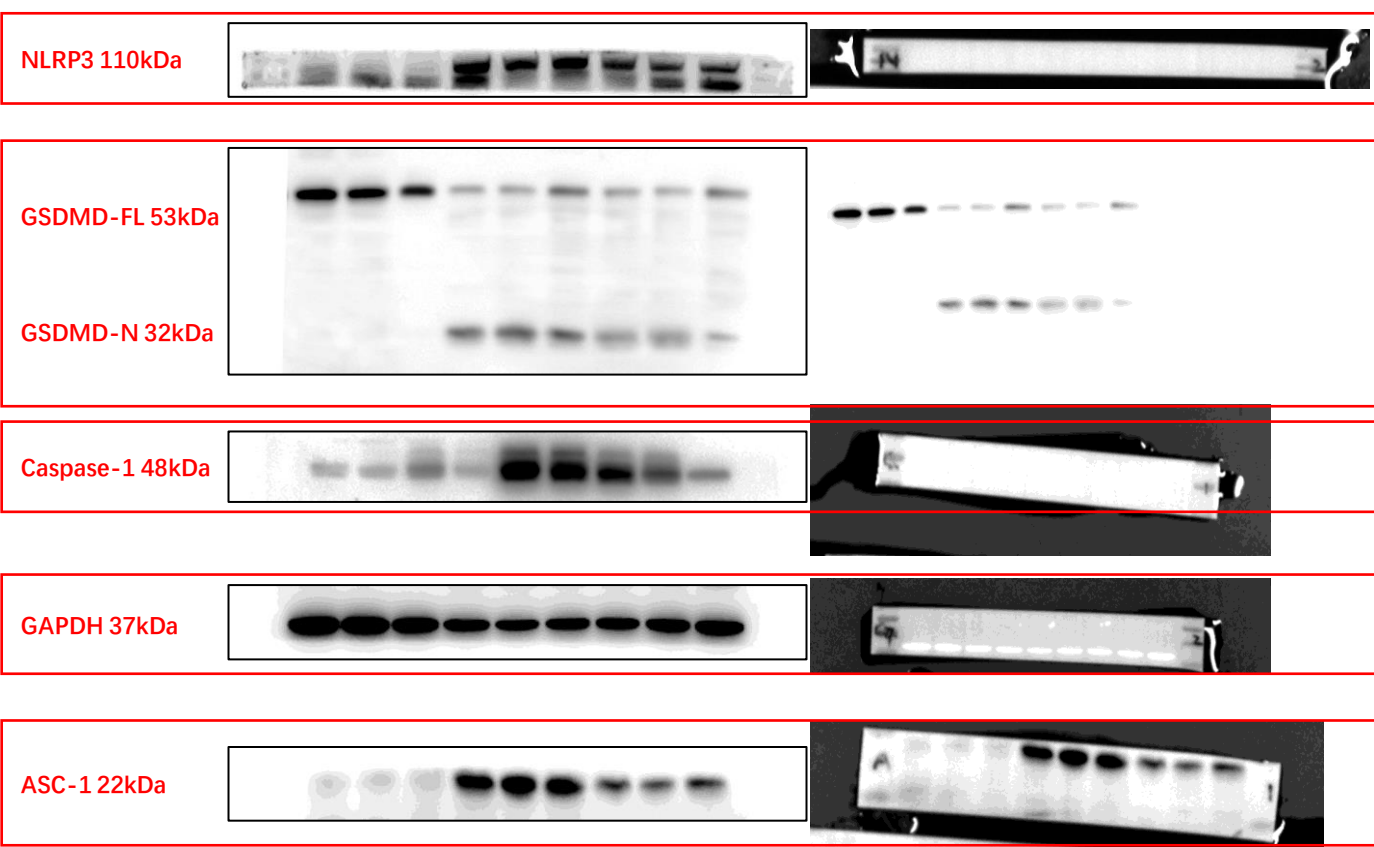

Supplement: Supplementary file 8 — Original Data File [file 41419_2022_4910_MOESM8_ESM.pdf]
